# Precision of AI-Based Lower Limb Assessment in Children and Adolescents with Open Growth Plates

**DOI:** 10.3390/diagnostics16193134

**Published:** 2026-09-26

**Authors:** Samuel Hohenberger, Monika Herten, Lea Alexandra Simmler, Cedric Rubenthaler, Johannes Haubold, Bastian Mester, Heinz-Lothar Meyer, Manuel Burggraf, Marcel Dudda, Christina Polan

**Affiliations:** 1 Department of Trauma, Hand and Reconstructive Surgery, University Hospital Essen, 45147 Essen, Germanybastian.mester@uk-essen.de (B.M.); heinz-lothar.meyer@uk-essen.de (H.-L.M.); marcel.dudda@uk-essen.de (M.D.); christina.polan@uk-essen.de (C.P.); 2 Institute for Artificial Intelligence in Medicine, University Hospital Essen, Girardetstraße 2, 45131 Essen, Germany; johannes.haubold@uk-essen.de; 3 Institute of Diagnostic and Interventional Radiology and Neuroradiology, University Hospital Essen, 45147 Essen, Germany; 4 Department of Orthopedics, Trauma, Hand- and Footsurgery, GFO Kliniken Mettmann-Süd, 40724 Hilden, Germany; manuel.burggraf@gfo-kliniken-mettmann-sued.de; 5 Department of Orthopedics and Trauma Surgery, BG Klinikum Duisburg, 47249 Duisburg, Germany

**Keywords:** artificial intelligence, pediatric orthopedics, automated radiographic measurements, full-length standing radiography, lower limb alignment, leg length discrepancy, mechanical leg angles, mechanical axis deviation

## Abstract

**Background/Objectives:** X-rays are the most reliable and widely available imaging modality for diagnosing deformities of the lower extremities in paediatric orthopaedic patients. This raises the question of whether AI-generated analysis of key parameters is comparable to manual measurement. **Methods:** This retrospective, single-center observational study included patients with radiographically confirmed open growth plates at a Level One university hospital in Germany between 2005 and 2022. Using the quality criteria (full-leg weight-bearing radiograph with the patella centered, showing both legs with the upper ankle joint and pelvis, in a bipedal, hip-width stance), 325 X-rays were evaluated for leg length measurement and 269 X-rays for leg angle measurement. For each parameter, measurements were taken manually by a specialist and compared with the results from the AI software BoneMetrics developed by Gleamer (Paris, France). The age range of the patients at the time of examination was for length measurements between 3.4 and 17.8 years; 59.4% were male (mean 12.5 ± 3.0 years) and 40.6% were female (mean 10.8 ± 2.8 years). For angle measurements, the study included a cohort ranging in age from 10.1 to 17.7 years; 58% were male (mean age 13.6 ± 1.7 years) and 42% were female (mean age 12.5 ± 1.4). **Results:** For leg length measurements, the mean values of the AI-based measurements for the femur were 1.4% (5.6 mm) lower on both sides than those of the manual measurement; however, they were higher on both sides for the tibia (6.5%, 21.7 mm) and total leg-length (5.3%, 40.4 mm). The correlation of leg length measurements showed no differences between the right and left sides. For all parameters (femur, tibia, and total length), the measurement results from the two methods correlated excellently, with an intraclass correlation coefficient (ICC) > 0.75. The combined effect of excellent ICC values, coupled with systematic deviations in tibial- and total leg length suggests that high reliability does not necessarily mean interchangeability of the two methods. For leg angle measurements, the percentage differences ranged from 0 to a maximum of 1.3%. The joint line convergence angle (JLCA) was an exception on both sides, with very high percentage differences of 27.2% on the right and 50.5% on the left. The mechanical axial deviation (MAD) showed significantly higher values on both sides for the AI measurement compared to the manual measurement (*p* < 0.001). The percentage difference was 9.2% on the right and 5.4% on the left. The ICC showed excellent values of >0.75 for 7 out of 10 angles and good values of 0.71, but poor values of 0.08 and 0.146 for the left and right JLCA, respectively. **Conclusions:** Manual measurement remains vital for the diagnosis of axial deformities in children and adolescents. Given some promising results, it is expected that, through continuous training, AI will eventually be able to match the radiological values determined manually.

## 1. Introduction

The physiological growth of the lower extremities during childhood and adolescence involves continuous axial and longitudinal changes [1,2]. While early childhood varus and valgus deformities usually resolve spontaneously [3], disorders of the growth plates, congenital anomalies or acquired conditions can lead to persistent leg-length discrepancies and axial malalignments [4]. Early differentiation between physiological and pathological changes is crucial to prevent long-term functional limitations [5].

In skeletally immature patients, growth modulation via temporary hemi-epiphysiodesis is an established minimally invasive and reversible alternative to corrective osteotomies [6,7]. However, treatment success depends largely on precise preoperative deformity analysis and correct assessment of the remaining growth potential [8]. Errors in the localization or quantification of the deformity can lead to under- or over-correction, significantly compromising clinical outcomes [9,10].

Standardized analysis of lower limb deformities relies on weight-bearing full-leg standing radiographs to measure leg lengths (tibia, femur and total leg length) and mechanical angular parameters described by Paley [11,12]. These measurements form the basis for preoperative planning and postoperative follow-up. Despite established digital planning software, manual measurement remains time-consuming, depends on the examiner’s experience and is prone to intra- and interobserver variability [13].

Consequently, the use of artificial intelligence (AI) and deep learning algorithms is increasingly integrated into orthopaedics to automate the detection of anatomical landmarks and standardized calculation of radiological parameters within seconds [14]. While initial studies demonstrate high agreement with manual measurements and substantial time savings, the current data primarily derive from adults or small paediatric cohorts [15,16,17,18]. Open growth plates and age-dependent anatomy pose special challenges for automated measurement systems, and data on AI reliability in children remain scarce.

Therefore, the aim of this study was to investigate the accuracy and reliability of an AI-based software for automated measurement of leg lengths and Paley angle measurements on full-leg radiographs in children and adolescents with open growth plates. Automatically generated measurements were compared with established manual measurements. In addition, the study analyzed whether patient age or gender influences the agreement between both methods.

## 2. Materials and Methods

### 2.1. Study Design and Data Collection

This retrospective, single-center observational study evaluated paediatric and adolescent patients with open growth plates who underwent weight-bearing long-leg radiography as part of routine clinical care between January 2005 and December 2022. Radiographs included both internal views and imported external sources. The institutional ethics committee approved the study (23-11455-BO), and the requirement for parental informed consent was waived due to complete data anonymization.

Demographic and clinical data, including age at radiography, gender, growth plate status, and radiographic calibration method (automated via a measuring sphere or manual via a ruler) were extracted from the medical record system Medico (CompuGroup Medical SE & Co. KGaA (CGM), Koblenz, Germany).

### 2.2. Selection Criteria and Image Analysis

Inclusion required a standardized full weight-bearing radiograph with a centered patella, vizualizing both legs with the upper ankle joint and pelvis, in a bipedal, hip-width stand. Exclusion criteria comprised severe leg deformities, as well as chronic diseases of the bone and prior hip or knee replacements. Based on these quality criteria, 325 radiographs were evaluated for leg length and 269 for leg angle measurement (Figure 1).

Manual measurements by a specialist were compared with the results from the AI software BoneMetrics (version 2.5.0.1) developed by Gleamer (Paris, France). The final cohort for length measurements ranged in age from 3.4 to 17.8 years; 59.4% were male (mean age 12.5 ± 3.0 years) and 40.6% were female (mean age 10.8 ± 2.8 years). For angle measurements, the study included a cohort ranging in age from 10.1 to 17.7 years; 58% were male (mean age 13.6 ± 1.7 years) and 42% were female (mean age 12.5 ± 1.4). The age range differed between the two analyses due to BoneMetrics software-specific age restrictions.

### 2.3. Manual Measurements

Manual measurements were performed using TraumaCad software (tCAD, Brainlab, Munich, Germany, version 2024) based on manually placed anatomical landmarks to calculate leg lengths and angles. All radiographs fulfilling the quality criteria were measured by a trained investigator under the direct supervision of a senior paediatric orthopaedist and a senior radiologist. The primary manual measurement was performed without knowledge of the AI-generated results and served as a reference standard for comparison with the automated measurements. Whenever we identified a significant discrepancy between manual and AI-based measurement, the corresponding manual measurement was repeated by a senior paediatric orthopaedist to rule out obvious measurement errors or errors in the placement of the landmarks before it was included in the final dataset. During this quality control step, blinding to the AI result was no longer maintained. This approach thus constitutes a quality control step and was not an independent, repeated measurement or a formal reliability assessment.

### 2.4. AI-Based Measurements

Automated radiographic analysis was performed using BoneMetrics software (Gleamer, Paris, France). This deep learning-based tool automatically detects anatomical landmarks—including the center of the femoral head, femoral condyles, tibial plateau and distal tibial joint—to calculate lower extremity length and angle parameters. The software has been previously validated for the automated measurement of full-leg standing radiographs (KBAs) [17,19]. The updated software (July 2025) used in this study automatically detects landmarks; measures the length of the tibia, the femur and the total leg; determines the mechanical leg axis; and calculates the Paley angle. All automatically generated measurement points and trajectories were overlaid onto separate images and reviewed using the institutional universal viewer (Centricity; GE HealthCare, Düsseldorf, Germany).

### 2.5. Anatomical Definitions and Leg Length Metrics

#### 2.5.1. Leg Length Parameters

Leg length was defined using three anatomical landmarks: the center of the femoral head, the center of the knee, and the lowest point of the tibial plafond. The knee center was defined as the projection of the center of the intracondylar notch onto a line tangent to the distal femoral condyles.

The total leg length was measured by drawing a line from the center of the femoral head to the lowest point of the tibial plafond. The length of the femur was measured from the center of the femoral head to the knee center, and the length of the tibia was measured as the distance from the knee center to the lowest point of the tibial plafond.

#### 2.5.2. Axial Parameters and Alignment

Frontal plane lower extremity alignment was quantified using the mechanical axis deviation (MAD), measured in millimeters. The mechanical axis was defined as the line projecting from the center of the femoral head to the center of the ankle joint. MAD was calculated as the perpendicular distance from this mechanical axis line to the knee joint center on standing full-leg radiographs (see Figure 2) [20].

#### 2.5.3. Radiographic Angle Measurements

Frontal plane knee alignment was further evaluated using specific mechanical and anatomical angles according to the Paley method [20] (Figure 3):

- Mechanical Lateral Distal Femoral Angle (mLPFA): The angle between a reference line, which begins at the exact center of the femoral head and ends at the tip of the greater trochanter, and the mechanical axis of the femur (the straight line connecting the center of the femoral head and the center of the knee joint).
- Mechanical Medial Proximal Tibial Angle (mMPTA): The medial angle formed between the mechanical axis of the tibia and the proximal tibial plateau tangent.
- Mechanical Lateral Distal Femoral Angle (mLDFA): The lateral angle between the anatomical axis of the femur and the distal femoral condylar tangent.
- Joint Line Convergence Angle (JLCA): The angle formed between the distal femoral condylar tangent and the proximal tibial plateau tangent, indexing knee joint laxity or cartilage loss.
- Mechanical Lateral Distal Tibial Angle (mLDTA): The angle formed by the intersection of the mechanical axis of the tibia (the line connecting the center of the tibial plateau and the center of the ankle joint surface) and the tangent line to the distal tibial articular surface.

### 2.6. Statistical Analysis

The unit of statistical analysis was one full-leg weight-bearing standing X-ray per patient. Though measurements were taken separately for the right and left limbs, the values from both sides were averaged at the patient level prior to the primary statistical analysis. Thus, each patient contributed one value per parameter to the analysis and the right and left limbs were not treated as independent samples. Consequently, no additional adjustment for within-patient correlation was necessary.

Statistical analyses were performed using SPSS (version 29.0; IBM Corp., Armonk, NY, USA) and Microsoft Excel (version 2021; Microsoft Corp., Redmond, WA, USA). Descriptive statistics were calculated as mean, median, standard deviation (SD), and minimum/maximum values. Normality of data distribution for measurement differences was assessed using the Kolmogorov–Smirnov and Shapiro–Wilk tests, respectively. Since data for leg lengths, angles, and mechanical axis deviation (MAD) were significantly not normally distributed, the Wilcoxon signed-rank test was applied for the pairwise comparison between manual and AI-based measurements. A *p*-value < 0.05 was considered statistically significant.

Agreement between the manual and AI-based metrics was illustrated using Bland–Altman analysis, created with GraphPad (Boston, MA, USA), to determine systematic bias and 95% limits of agreement (LoAs; calculated as bias ± 1.96 × SD). Although the data did not formally follow a normal distribution, a visual inspection of the histograms and Q-Q plots revealed no significant deviations from approximate symmetry or normality in the measurement differences. Given the large sample size and the known sensitivity of formal normality tests to minor deviations in large datasets, the conventional Bland–Altman approach was applied.

Reliability was quantified using the intraclass correlation coefficient (ICC) based on a two-way mixed-effects model evaluating absolute agreement for single measures [21].

To analyze the confounding influence of demographic variables, a general linear model (GLM) combined with analysis of variance (ANOVA) was applied to investigate the effects of sex and age on the measured parameters. Age was categorized into the following three groups: 3.38–11.1 years (*n* = 106); 11.15–13.15 years (*n* = 107); and 13.36–17.82 years (*n* = 112).

## 3. Results

### 3.1. Study Population

For the analysis of leg length measurements, 325 X-ray images of both the right and left legs were analyzed. Of these, 59.4% were male patients (*n* = 193) and 40.6% were female patients (*n* = 132). The average age of the boys was 12.5 ± 3 years and that of the girls was 10.8 ± 2.8 years. The age range of the patients was 3.4–17.8 years (Table 1).

To examine the mechanical leg angles, 269 X-rays were analyzed. Male patients accounted for 58% (*n* = 155) of the cohort, while 42% (*n* = 114) of the participants were girls. The average age of the boys was 13.6 ± 1.7 years and that of the girls was 12.5 ± 1.4 years. The age range of the patients was 10.1–17.7 years (Table 1).

### 3.2. Comparison Between Manually and AI-Generated Measurements

For all parameters examined, the measurement differences between manual and AI methods were statistically significant. Regarding limb length measurements, the largest discrepancies were found for tibial length and total leg length, with mean deviations of 21.7 mm (6.5%) and 40.4 mm (5.3%), respectively (Table 2). In contrast, angular parameters demonstrated high consistency between both methods, with mean deviations ranging between merely 0.12° and 1.04° (Table 3). The joint line congruence angle (JLCA) was a notable exception, exhibiting a high relative deviation of 37.7%, despite a minimal absolute mean difference of only 0.4°. For the mechanical axis deviation (MAD), a mean deviation of 0.91 mm was observed, corresponding to a relative difference of 7.4% (Table 4).

### 3.3. Bland–Altman Analysis

The Bland–Altman analyses of the leg length measurements revealed a significant bias for both tibial length and the total leg length (Figure 4). The mean difference between manual and AI-based tibial measurements was −21.7 mm, corresponding to a relative deviation of 6.5%. For total leg length, the average deviation amounted to −40.4 mm (5.3%). Both results indicate systematic overestimation by the AI. In contrast, the measurement discrepancies for femoral length were substantially smaller, demonstrating a bias of only +5.6 mm (1.4%) compared to the other two length parameters and indicating underestimation by AI.

Bland–Altman analysis of the angle measurements demonstrated minimal systematic differences between manual and AI-generated angular measurements (Figure 5). Overall, AI measurements were slightly larger than manual ones, with a mean difference of 0.76° (relative deviation 0.85%). Parameter-specific biases were as follows (mean difference of both sides): mLPFA: −0.4° (0.9%); mLDFA: −0.7° (0.1%); mMPTA: −0.7° (1.2%); mLDTA: 0.9° (0.60%); JLCA: 0.4° (37.7%) and MAD: −0.8 (7.4%).

To further evaluate the practical magnitude of our measurement differences, we compared the values with previously published standard error of measurement (SEM_95%_) values for pediatric long leg radiography [13]. The proportions of AI measurements falling within these established human interobserver error ranges were 68.8% for mLPFA, 88.1% for mLDFA, 51.3% for mMPTA, 85.1% for mLDTA, 67.3% for JLCA, and 69.5% for MAD. For length measurements, 41.5% of femoral measurements were within the published SEM_95%_ reference range, whereas only 0.3% of tibial measurements met this criterion. No published SEM_95%_ reference value was available for total leg length.

### 3.4. Reproducibility of Measurement Results

Measurements of femoral, tibial, and total leg length demonstrated excellent agreement between manual and AI-based measurements (Figure 6). However, both tibia and total leg length simultaneously exhibit systematic biases of −21.7 mm and −40.4 mm, respectively, demonstrating that high reliability does not imply the absence of systematic biases.

For four out of five angular parameters, the intraclass correlation coefficient (ICC) indicated excellent agreement. The joint line congruence angle (JLCA) was a notable exception, showing poor agreement bilaterally with an ICC value of 0.154 (Figure 6).

### 3.5. Influence of Age and Sex

For leg angle measurement, the general linear model analysis showed that neither gender nor age group influenced the difference between the manual measurement and the AI-based measurement. However, the differences in measurement results for the right femur (*p* = 0.047) and total leg length (*p* = 0.037) were smaller in the youngest age group than in the oldest group.

Similarly, for the analysis of leg angles, the study found no consistent influence of age or gender, with the exception of an isolated age-related effect on mLDTA. There was a statistically significant effect of age on the measurement results for the left mLDTA (*p* = 0.044) and a borderline significance for the right mLDTA (*p* = 0.053).

## 4. Discussion

### 4.1. Key Results

The present study demonstrates that the BoneMetrics AI software accurately measures mechanical leg angles on anteroposterior (a.p.) radiographs. The only exception was the joint line convergence angle (JLCA) on both the sides, which exhibited significant deviations and poor reproducibility.

While leg length measurements showed consistently excellent reproducibility, significant systematical deviation occurred in tibial and total leg lengths. No correlation between the measurement differences and age or gender was observed. Thus, the results were promising; however, improvements to the measurements are considered necessary before AI can be relied upon in routine medical diagnostics.

### 4.2. Limitations and Interpretation

When evaluating discrepancies between AI-based and manual measurements, intra- and inter-observer variability of human raters must be considered. Therefore, to assess the accuracy of automated measurements, AI-to-human differences should ideally fall within the range of standard inter-observer variability.

Although the use of AI is already well-established in many medical fields, the literature regarding pediatric orthopedics is limited. Regarding reproducibility, our results align with Moon et al., confirming mostly excellent intraclass correlation coefficients (>0.90) for most angles and leg lengths [22]. Furthermore, similar to Hoffmann et al., our analysis demonstrates a positive trend, establishing high agreement for most angular parameters [15]. A key strength of our study is the large cohort of 269 and 325 X-rays, respectively, from pediatric orthopedic patients, which significantly exceeds the sample sizes of earlier publications—such as that by Van der Lelij et al. [16].

AI-based measurements of the JLCA showed the greatest limitations, consistently underestimating values and exhibiting poor reproducibility compared to manual measurement, despite identical axis definitions. Factors contributing to these discrepancies could include the narrow physiological range of the angle (0–2°), challenges in identifying the joint lines due to increased soft tissue in the immature skeleton, and projection artefacts. To date, no other pediatric orthopedic studies evaluating the JLCA with this or alternative AI software is available for direct comparison. However, our findings align with the adult patient literature; Stotter et al. similarly demonstrated that manual JLCA measurements are highly susceptible to intra- and inter-observer variability [23]. Given that an increased JLCA directly correlates with the development and progression of intra-articular knee osteoarthritis, algorithmic refinement and targeted training of AI models using paediatric knee joints are of high clinical relevance [23,24]. With our study, we cannot determine with certainty the cause of the poor agreement of the JLCA. Since the software does not provide access to the internal landmark coordinates or confidence metrics, these mechanisms could not be analyzed separately. We therefore consider the observed JLCA error to be multifunctional, with a narrow physiological angle range, difficult joint line distinction, projection effects and potential errors in landmark localization all representing plausible causes.

Regarding leg length measurement, greater AI measurement discrepancies are observed for the tibia and total leg length compared to the femur. This variance likely stems from the femur possessing distinct and easily identifiable anatomical landmarks. In contrast, the tibia features complex structures, particularly distally at the tibiofibular joint. In this region, confounding factors such as fibular overlap, projection artefacts, and rotational variations can compromise the precise algorithmic placement of measurement points, adversely affecting tibial and, consequently, total leg length measurement [25]. In addition, because the BoneMetrics software for length measurements was evaluated on a pediatric cohort including children as young as 3 years old, the system faces unique challenges due to immature skeletal structures that differ substantially from adult anatomy [26].

In contrast, the consistent deviations in tibial length and total leg length measurements appear to be less easily explained by standard observer variability alone. The mean deviation of 5.7 mm for the femoral length measurements was relatively close to previously reported measurement errors. However, the deviations of −21.7 mm for tibial length and −40.4 mm for total leg length were considerably larger. A study published by Zheng et al. demonstrated a deep learning algorithm achieving a mean absolute error of approx. 4.5 mm in measurements of femur, tibia and total leg length in a pediatric cohort [27]. Meanwhile, they showed a significant reduction in measurement time compared to trained radiologists.

Similarly, Larson et al. reported correlations of over 0.99 between AI-generated and radiologist-determined femur, tibia and total leg length measurements, with mean errors below 1% [26].

Compared to these results, the deviations observed in our study were an 6.5% increase for tibia length and a 5.3% increase for total leg length in AI measurement and suggest a systematic rather than a purely random error. Possible explanations include differences in the definition of landmarks, radiological calibration or algorithm-specific localization of reference points, especially at the knee and ankle joints.

Consequently, excellent ICC values alone should not be interpreted as evidence of interchangeability, as a method can correlate strongly with the reference standard and yet still produce a consistent systematic bias [28].

The comparatively small discrepancy in femur length, in contrast to the consistent overestimation of tibia length and total leg length is another remarkable observation. One plausible explanation for the greater discrepancy in total leg length is that different combinations of landmarks are used for total leg length and bone-specific measurements: total leg length extends across the radiographic knee joint region, while femur and tibia lengths terminate at separate bone-specific landmarks. However, since both systems were designed to use the same anatomical landmarks, this cannot sufficiently explain the consistent tibial distortion and instead suggests a systematic difference in the localization of landmarks or in the calculation of length measurements. In case of radiological calibration, a global magnification error would likely affect all linear measurements in a similar direction and by the same ratio. We therefore consider parameter-specific differences in the localization or orientation points—particularly at the center of the knee joint and the distal tibial plateau—as well as discrepancies in the internal calculation of segment lengths to be more plausible causes. Since BoneMetrics is a proprietary system, the exact localization of the landmarks and the internal scaling method cannot be reconstructed.

Projection-related scaling could also play a role if the systems use different calibration methods, as the distance between the patient and the detector, the position of the calibration marker in the sagittal plane, and the radiographic imaging system can influence linear measurements.

Nevertheless, the comparable TraumaCad results (manual/automatic) argue against manual calibration as the sole cause. Given the long data collection period from 2005 to 2022, changes in detector technology, image registration, or data collection protocols may have caused additional variability. We therefore cannot unequivocally attribute the observed bias to a single mechanism.

The distinction between statistical significance and clinical relevance is of great importance for the interpretation of the present results. Due to the relatively large study sample size, even minor systematic differences between manual and AI-based measurements reach statistical significance. However, some of the observed angular differences were of an order of magnitude comparable to the intrinsic variability of conventional radiological measurements. In a pediatric cohort in which a deformity analysis was also performed, Braun et al. reported standard measurement errors (SEM_95%_) of approx. 4.53 mm for the femur length, 3.12 mm for tibia length, 1.69° for the mLPFA, 1.06° for the mLDFA, 1.29° for the mMPTA, 2.17° for the mLDTA, 1.38° for the JLCA and 3.31 mm for the MAD [13]. The proportion of AI measurements falling within Braun et al.’s previously reported human interobserver measurement-error ranges was particularly high for mLDFA (88.1%) and mLDTA (85.1%), while 68.8% of mLPFA, 51.3% of mMPTA, and 69.5% of MAD measurements were within the respective SEM_95%_ reference ranges. In contrast, only 41.5% of femoral length measurements and 0.3% of tibial length measurements fell within the corresponding reference ranges, further supporting a relevant systematic discrepancy in length measurements, particularly for the tibia. Although 67.3% of JLCA measurements were within the published SEM_95%_ range, poor ICC and high manual vs. AI deviation indicate that this parameter remains insufficiently reliable. Taken together, these findings suggest that the extent of disagreement for several angular parameters is comparable to the measurement uncertainty observed between human raters, whereas the systematic discrepancies in tibial length remain substantially greater. Nevertheless, the SEM_95%_ values reported by Braun et al. represent human interobserver measurement uncertainty rather than universally established clinical acceptance thresholds and should therefore not be interpreted as proof of clinical interchangeability [13]. The JLCA remains a notable exception, and not only in our study [29]. Interestingly, Braun et al. also identified the JLCA as the least robust parameter in pediatric/adolescent full-leg standing x-rays and reported a proportional error of 138% [13].

This suggests that the low degree of agreement we observed may not be due exclusively to an algorithmic limitation of the AI, but also to the inherent difficulty of defining a very small joint-related angle in a reproducible manner.

Another source of uncertainty is patient positioning during X-ray imaging. In a three-dimensional simulation study, Brunner et al. demonstrated that the rotation and flexion of the lower extremity can significantly alter the measurements derived from two-dimensional full-leg standing X-rays, including MAD, mMPTA and mLDFA [30]. A combination of rotation and knee flexion led to particularly pronounced deviations. Since the manual and automated measurements in this study were derived from the same X-ray image, systematic differences between the two methods cannot be directly explained by positioning errors. Nevertheless, projection-related changes in the appearance of anatomical landmarks can influence how a human observer and an AI-based landmark detection algorithm identify the same structure. This could be particularly relevant at the femoral condyles, the tibial plateau and the distal tibia, where overlapping structures and rotational changes can alter the visible bone contour [31]. Focusing on this aspect in future AI systems could therefore not only lead to improved landmark detection, but could also, through automated image quality assessment, detect incorrect rotations, insufficient knee extension, or improper positioning before measurements are taken [32].

The influence of skeletal maturity must also be considered. The largely absent correlation between demographic parameters and measurement differences suggests that the algorithm yields consistent results within the age range studied. However, this finding should not be interpreted as evidence of unrestricted applicability throughout childhood. While the cohort for leg length measurements include patients aged 3.4 to 17.8 years, the patients for whom angle measurements were performed were between 10.1 and 17.7 years old. Given that BoneMetrics is not currently approved for angle analysis in children under 10 years of age, our findings regarding angle measurements should not be generalized to younger, immature children. This is particularly relevant because automated measurement of mechanical leg angles in younger children, in whom open growth plates, incomplete ossification, and age-related changes in epiphyseal morphology can further complicate the identification of landmarks [33]. Consequently, the present study validates AI-based angle measurements only for children aged 10 years and older with open growth plates, while the algorithm’s performance in younger children remains unknown and requires separate validation.

Another limitation is the lack of formal analyses of intra- and inter-observer reliability for the manual reference measurements we performed. These measurements were conducted by a trained examiner under the supervision of a pediatric orthopedic specialist and re-evaluated in cases of significant discrepancies between the manual and AI results; however, this quality control procedure does not replace independent, repeated measurements. To provide additional context, the observed differences (as previously described) between manual and AI-based measurements were therefore compared with published data on measurement uncertainty in pediatric interobserver comparisons [13]; however, this external reference value cannot replace study-specific reliability data.

### 4.3. Generalizability

As previously described, deep learning studies have shown that automated analysis of the leg parameters can significantly reduce measurement time while maintaining high overall agreement with manual measurements [27,34]; however, recent studies have also emphasized that joint-level parameters remain more challenging than global alignment parameters, consistent with our findings (JLCA deviation of 37.7%) [15,35,36]. Taken together, these results suggest that, at this stage, AI should be used as an aid to measurement rather than as a standalone diagnostic system.

## 5. Conclusions

The AI-based, automated analysis showed promising agreement with manual reference measurements for several angular parameters, particularly for mLDFA and mLDTA, where a high proportion of the measured values fell within the previously reported ranges of inter-observer measurement uncertainty in humans [13]. In contrast, the systematic deviations observed in tibial and total leg length, as well as the poor reproducibility of the JLCA, suggest that these parameters require particular caution and expert review. Therefore, automated measurements can support and standardize radiographic assessment in pediatric orthopedics; however, they should currently be regarded as an additional tool rather than a substitute for expert judgment. Further prospective, multicenter validation across different AI-based systems, pediatric age groups, and deformity patterns will be necessary before fully automated analysis can replace manual measurement in the daily pediatric orthopedic routine [37].

## Figures and Tables

**Figure 1:**
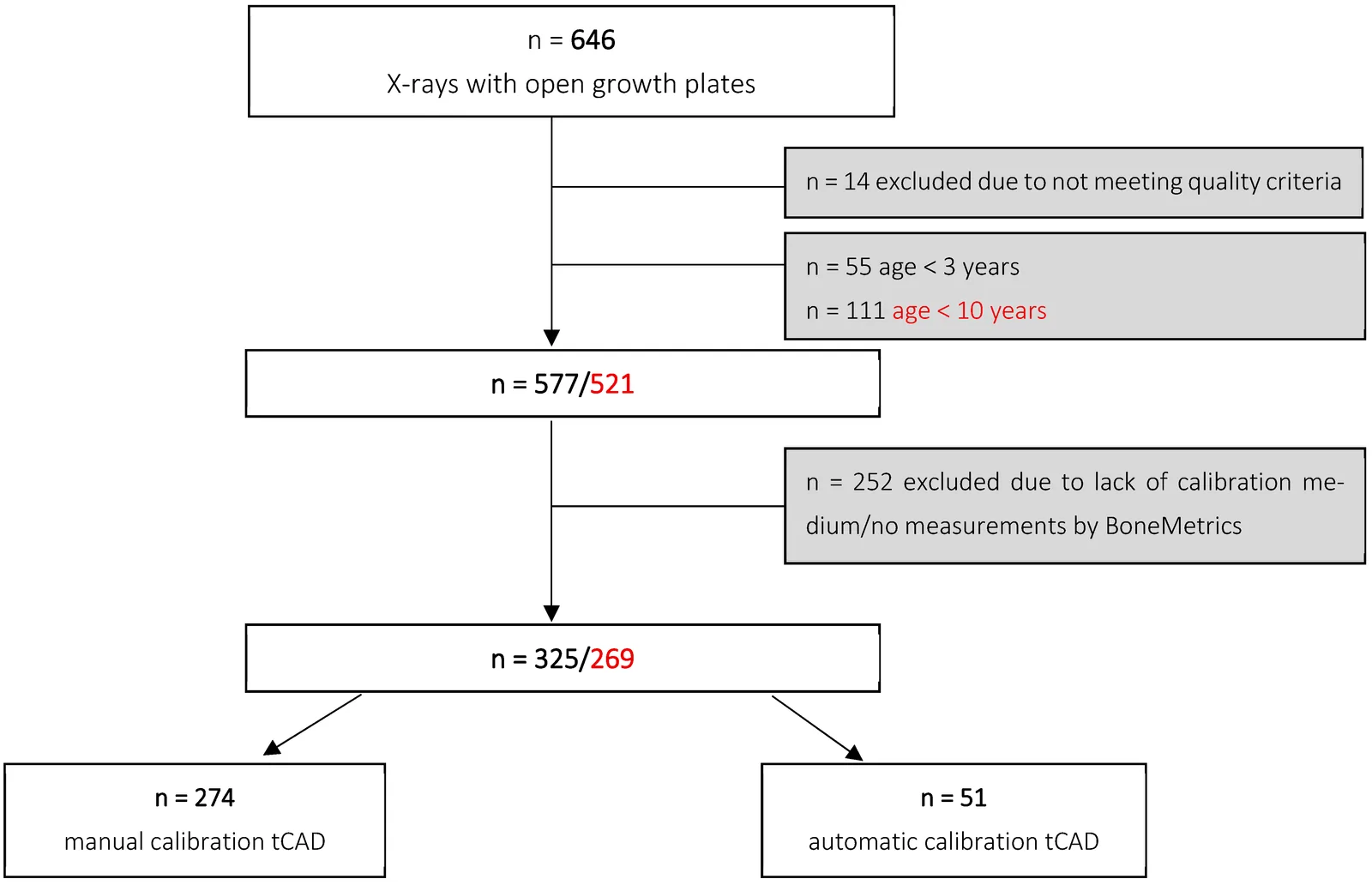
Flowchart illustrating the data collection process. The complete set of X-ray images is shown, along with the images we excluded and the corresponding exclusion criteria. The images are divided into manually and automatically calibrated groups, as well as into leg length and leg angle measurements (shown in red).

**Figure 2:**
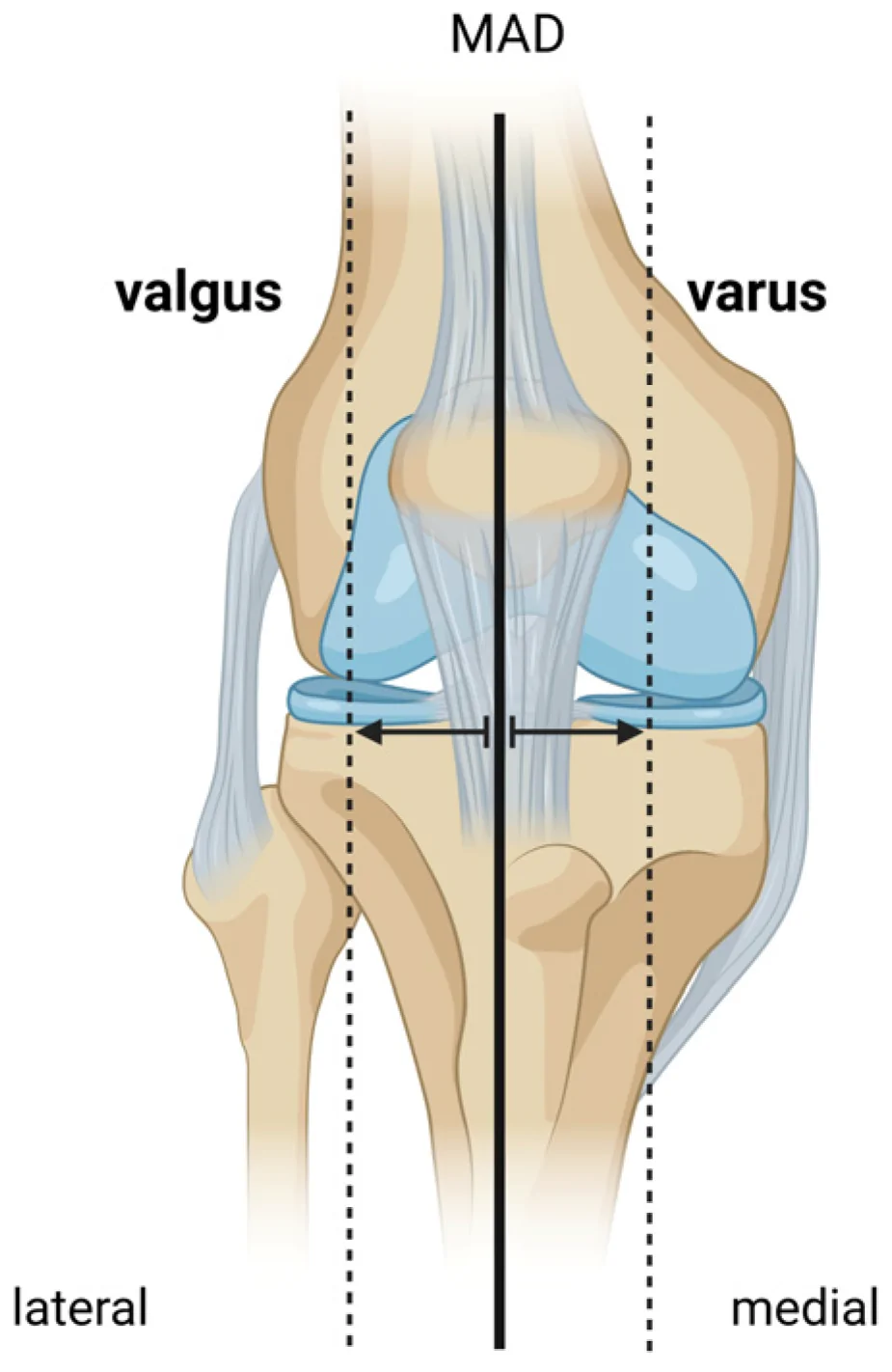
Illustration of mechanical axis deviation (MAD); created with BioRender.

**Figure 3:**
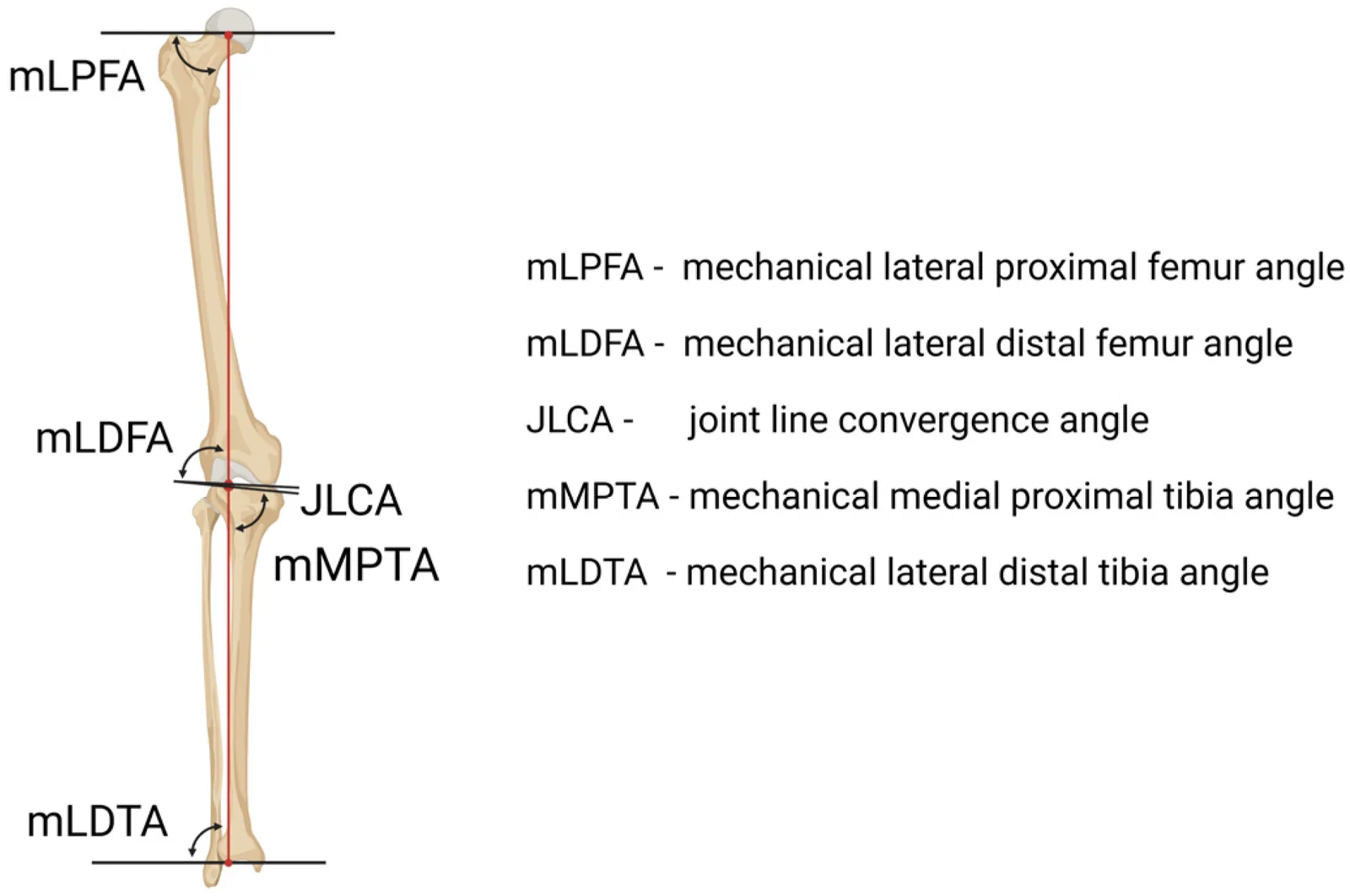
Illustration and labeling of the leg angles studied; created with BioRender.

**Figure 4:**
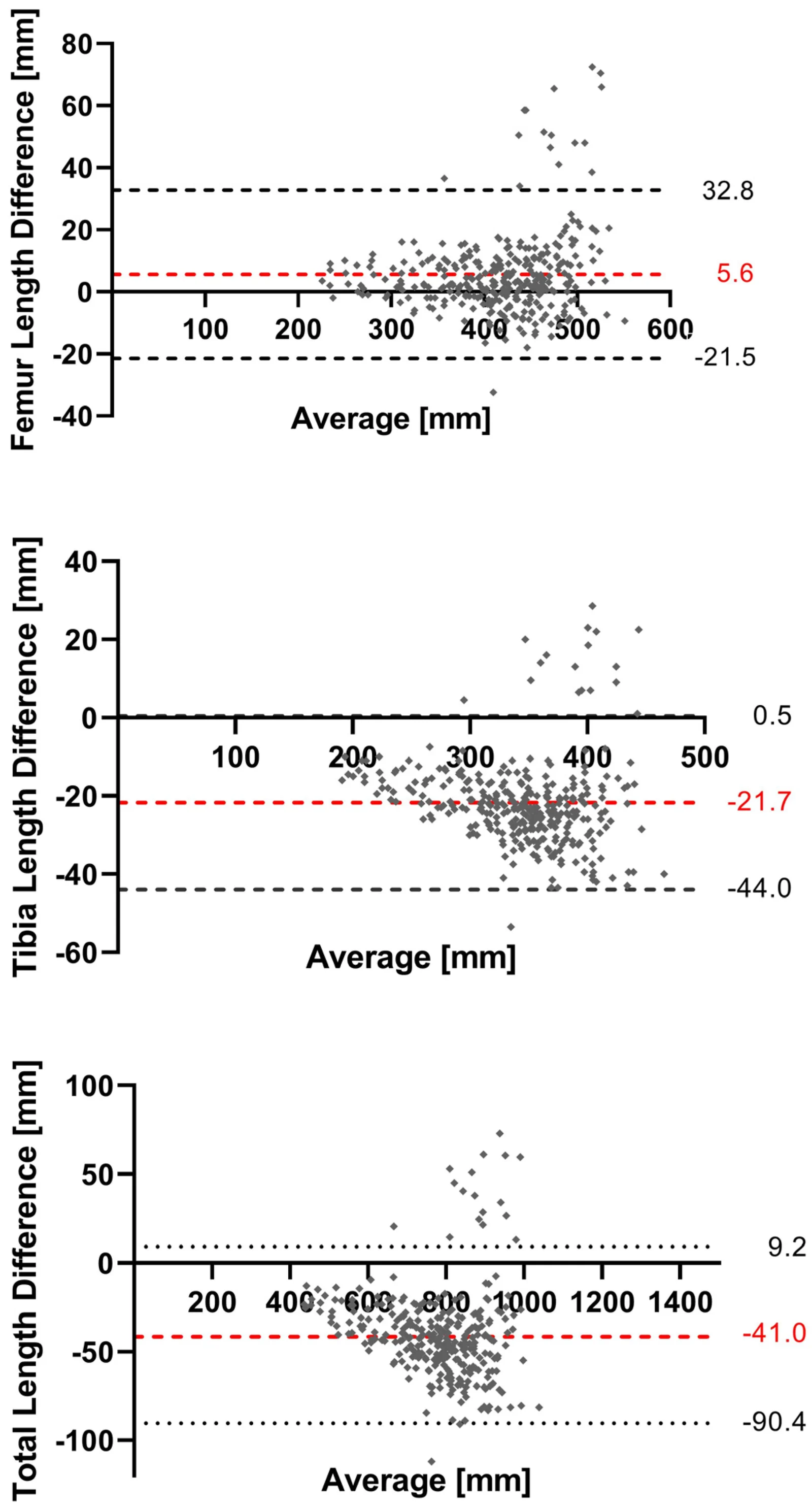
Comparison of manually and AI-assisted leg length measurements in conventional radiological examinations of the leg axis, presented in Bland–Altman plots. The black dots show the differences between the measurement methods for each X-ray image. The mean difference between the two measurement methods is shown as a red dashed line. The black dashed lines indicate the upper and lower limits of variation. They are calculated as the mean ±1.96 times the standard deviation of the differences.

**Figure 5:**
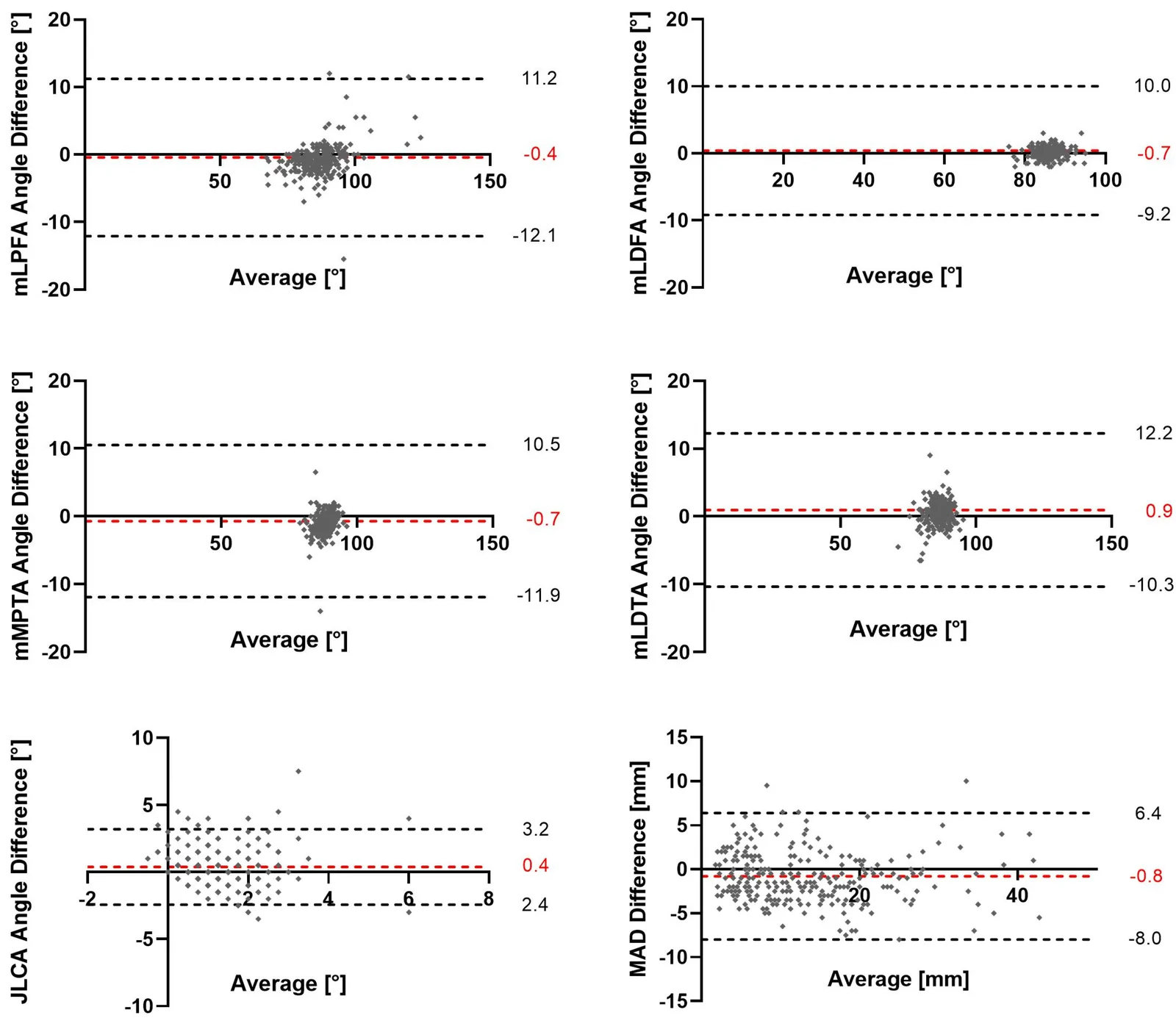
Comparison of manually and AI-assisted mechanical leg angle measurements, as well as mechanical axial deviation (MAD), in conventional radiological examinations of the leg axis, presented in Bland–Altman plots. The black dots indicate the differences between the measurement methods for each X-ray image. The mean difference between the two measurement methods is shown as a red dashed line. The black dashed lines indicate the upper and lower limits of variation. They are calculated as the mean ±1.96 times the standard deviation of the differences.

**Figure 6:**
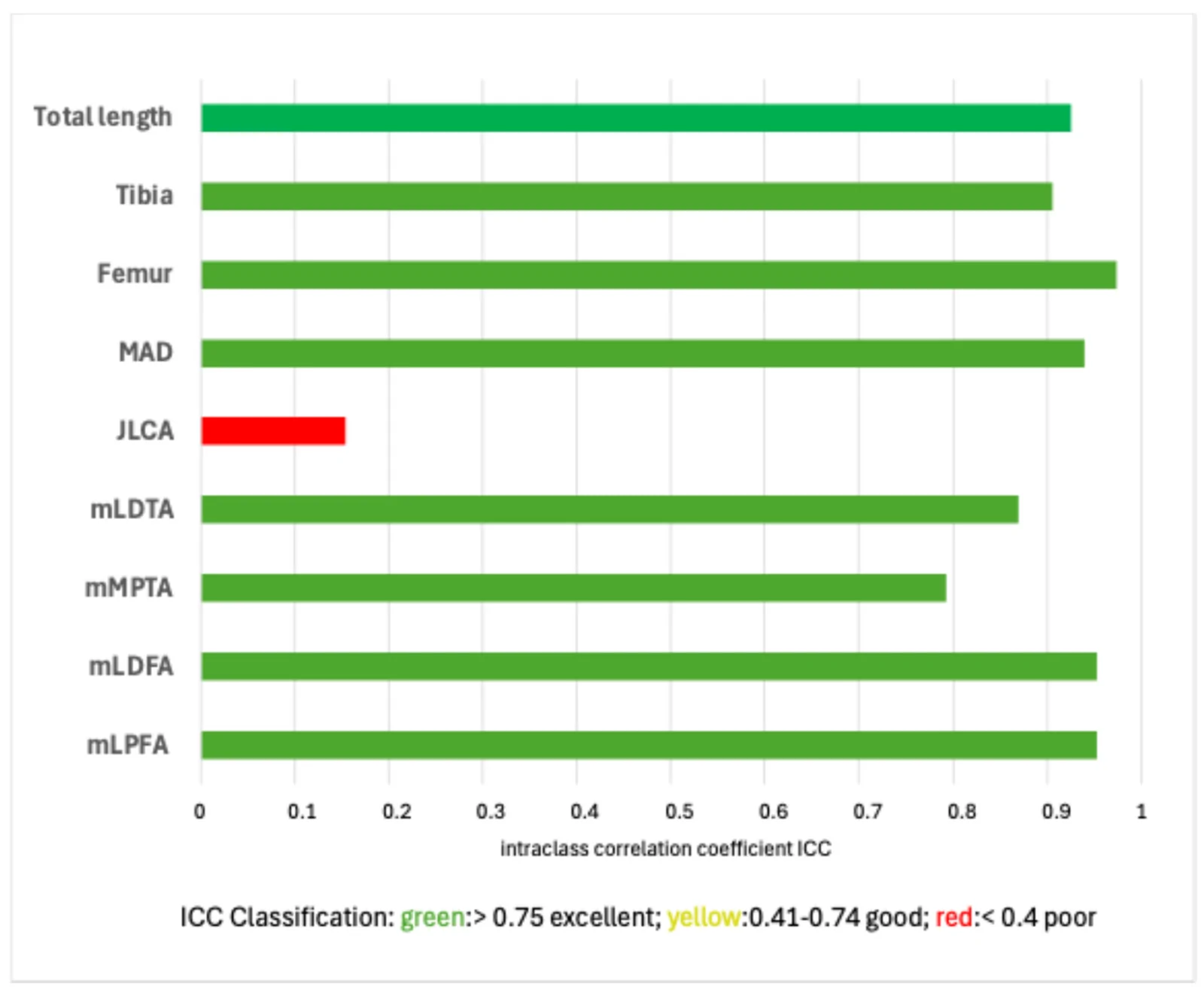
Intraclass correlation coefficient (ICC) for leg length and leg angles measured manually versus by AI. The figure shows the correlation between manual and automated angle measurements from *n* = 269 X-ray images for leg angle measurements and *n* = 325 X-ray images for leg length measurements. MAD: mechanical axis deviation; JLCA: joint line convergence angle; mLDTA: mechanical lateral distal tibial angle; mMPTA: mechanical medial proximal tibial angle; mLDFA: mechanical lateral distal femoral angle; mLPFA: mechanical lateral proximal femoral angle.

**Table 1:** Patient characteristics. Patients under the age of 10 were excluded for angle measurements (*). Patients under the age of 3 were excluded for length measurements. SD, standard deviation.

Number of X-rays included	325/269 *	
Gender	*N*	%
Male	193/155 *	59.4%/58.0% *
Female	132/114 *	40.6%/42% *
Age in years	length	angle
Male	12.5 ± 3	13.6 ± 1.7
Female	10.8 ± 2.8	10.1–17.7
Length measurements in cm, mean ± SD		
Male	12.5 ± 3.0	
Female	10.8 ± 2.8	
Angle measurements in °, mean ± SD		
Male	13.6 ± 1.7	
Female	12.5 ± 1.4	

**Table 2:** Manually and AI-generated length measurements; femur (femoral length), tibia (tibial length), total (total leg length).

Length	*n*	Manual Mean ± SD [mm]	AI Mean ± SD [mm]	Difference (Manual–AI) Mean ± SD [mm]	Difference [%]	*p*-Value
Femur	325	418.5 ± 66.1	412.9 ± 64.4	5.6 ± 13.8	1.4	<0.001
Tibia	325	332.9 ± 53.7	354.6 ± 55.0	−21.7 ± 11.3	6.5	<0.001
Total	325	757.8 ± 119.6	798.3 ± 122.1	−40.4 ± 25.4	5.3	<0.001

**Table 3:** Manually and AI-generated angle measurements: mLPFA (mechanical lateral proximal femoral angle), mLDFA (mechanical lateral distal femoral angle), mMPTA (mechanical medial proximal tibia angle), mLDTA (mechanical lateral distal tibia angle), JLCA (joint line convergence angle).

Angle	*n*	Manual Mean ± SD [°]	AI Mean ± SD [°]	Difference (Manual–AI) Mean ± SD [°]	Difference [%]	*p*-Value
mLPFA	269	86.22 ± 8.31	86.98 ± 7.41	−0.76 ± 2.30	0.9	<0.001
mLDFA	269	85.80 ± 2.78	85.68 ± 2.67	0.12 ± 0.83	0.1	0.025
mMPTA	269	87.84 ± 3.04	88.88 ± 2.69	−1.04 ± 1.60	1.2	<0.001
mLDTA	269	86.70 ± 3.59	86.20 ± 3.36	0.50 ± 1.72	0.6	<0.001
JLCA	269	1.46 ± 1.08	1.06 ± 1.15	0.40 ± 1.44	37.7	<0.001

**Table 4:** Manually and AI-generated mechanical axis deviation (MAD) measurements.

Parameter	*n*	Manual Mean ± SD [mm]	AI Mean ± SD [mm]	Difference (Manual–AI) Mean ± SD [mm]	Difference [%]	*p*-Value
MAD	269	12.44 ± 8.66	13.36 ± 8.87	−0.91 ± 2.92	7.4	<0.001

## Data Availability

The datasets analysed during the current study are available from the corresponding author on reasonable request.

## Abbreviations

The following abbreviations are used in this manuscript:

AI	artificial intelligence
ANOVA	analysis of variance
GLM	general linear model
ICC	Intraclass Correlation Coefficient
JLCA	joint line convergence angle
LoA	limits of agreement
MAD:	mechanical axis deviation
mLDTA	mechanical lateral distal tibial angle
mLPFA	mechanical lateral proximal femoral angle
mMPTA	mechanical medial proximal tibial angle
tCAD	TraumaCad software
